# Both Restricted and Excessive Foetal Growth Increase the Odds of Failing Early Language Screening: A Register Study

**DOI:** 10.1111/apa.70712

**Published:** 2026-07-27

**Authors:** Anna Fäldt, Isabelle Liljekvist, Anton Dahlberg

**Affiliations:** ^1^ Department of Public Health and Caring Sciences/CHAP Uppsala University Uppsala Sweden

**Keywords:** foetal growth, language development, language screening, large for gestational age, small for gestational age

## Abstract

**Aim:**

To investigate whether birth size for gestational age is associated with failure at language screening in early childhood.

**Methods:**

This population‐based cohort study used linked national register data from Sweden. Children born between 2018 and 2023 who had undergone language screening at 2 years 4 months to 3 years 3 months of age were included (*n* = 194 637; 94 051 females, 100 586 males). Logistic regression estimated associations between birth size for gestational age and screening failure, with children born appropriate for gestational age as the reference group, adjusting for sex, preterm birth, parental education, maternal smoking, and parental country of birth.

**Results:**

Overall, 12% of children failed the language screening. Children born small for gestational age had higher odds of failure than appropriate for gestational age children (adjusted OR 1.42, 95% CI 1.30–1.54), as did children born large for gestational age (adjusted OR 1.24, 95% CI 1.15–1.33). Male sex, preterm birth, lower parental education, maternal smoking, and having two foreign‐born parents were each associated with screening failure.

**Conclusions:**

Both restricted and excessive foetal growth were associated with increased likelihood of failing language screening, suggesting that deviations from typical foetal growth patterns may affect language development.

## Summary

- Language development is shaped by multiple perinatal factors, yet how deviations in birth size relate to early language outcomes remains unclear.
- Both small and large for gestational age were associated with increased odds of failing language screening at 2.5–3 years, after adjusting for key covariates.
- Birth size for gestational age may be a clinically relevant indicator for child health services when identifying children needing closer monitoring of language development.

**Abbreviations**
AGA: appropriate for gestational age
DLD: developmental language disorder
LGA: large for gestational age
SGA: small for gestational age

Foetal growth patterns are closely associated with long‐term developmental outcomes [1, 2, 3]. These patterns are commonly categorized into small for gestational age (SGA) and large for gestational age (LGA) groups. The causes of being born SGA or LGA are multifactorial, including placental dysfunction, lifestyle‐related factors, maternal BMI, gestational diabetes, and genetic predisposition [4, 5]. SGA is commonly defined as a birth weight for gestational age below −2 standard deviations (SD), whereas LGA is defined as a birth weight for gestational age +2 SD [6, 7].

Most research has focused on the relationship between SGA status and later development, with reported associations with autism spectrum disorders, attention‐deficit/hyperactivity disorder, and language development [8, 9].

Developmental language disorder (DLD) is a common disorder, with a prevalence of 7% [10] in preschool children, impacting language comprehension and expression [11]. These difficulties lead to heightened risks for depression, anxiety, behavioural problems, aggression, and social withdrawal [12]. Preterm birth is associated with DLD [13] and several studies have demonstrated an association between SGA and language difficulties, including late language emergence [14, 15]. Smoking in utero is also associated with DLD [16], and so is gestational diabetes [17].

Boys more frequently fail early language screenings [18] and are overrepresented in clinical samples, although the prevalence of developmental language disorder is comparable in boys and girls when children's language abilities are formally assessed [16]. Further, socioeconomic status, including parental education, occupation, and income, is associated with language abilities [19, 20].

In Sweden, nearly all children undergo language screening between 2.5 and 3 years of age as part of the national child health service program. Two different screening methods are used, both of which have demonstrated satisfactory sensitivity and specificity and are designed to identify children with severe language disorder [21, 22].

The aim of the study was to investigate the association between being SGA or LGA and the outcome of the routine language screening.

## 1 Methods

### 1.1 Data Sources

The study was based on linked national register data. Information on sex, parental education, and parental country of birth was obtained from Statistics Sweden. Data on birth‐related characteristics, including gestational age, birthweight, small and large for gestational age, maternal smoking during pregnancy, and preterm birth, were obtained from the Swedish Medical Birth Register. Information on language screening outcomes was retrieved from the Swedish Child Health Services Quality Register. Individual‐level data from the registers were linked using the unique personal identity number assigned to all residents in Sweden.

Individual‐level informed consent was not required for this study, as it was based entirely on pseudonymised data from national registers. The use of register data for research without individual consent is permitted under Swedish law (Act on Ethical Review of Research Involving Humans, SFS 2003:460) when ethical approval has been obtained. Ethical approval was granted by the Ethics Review Authority of Sweden (dnr 2024‐03606‐01).

### 1.2 Variables

Birth size for gestational age was classified as SGA or LGA according to the Swedish foetal growth reference developed by Maršál et al. [7], based on birthweight in relation to gestational age and sex. Children born AGA served as the reference group in the analyses.

The outcome variable was the result of the routine language screening conducted within the Swedish child health services programme at approximately 2.5–3 years of age. Two scientifically validated methods are used within the programme: the Miniscalco method (administered from age 2 years 4 months to 2 years 8 months, using toy objects) and Uppsalamodellen (administered from age 2 years 6 months to 3 years 2 months, using standardized photographs). Both methods assess language comprehension, expressive vocabulary, sentence length, and articulation, and are designed to identify children with severe language difficulties [21, 22]. Screening results were coded as pass or fail according to the registration in the Swedish Child Health Services Quality Register. It should be noted that a fail result does not correspond directly to referral for speech and language pathology assessment. Rather, the outcome reflects the BVC nurse's registered screening result, which may be followed by referral or repeated screening, with additional parental support and guidance provided as indicated.

Potential confounding variables were selected a priori based on previous literature on perinatal factors and early child development. These included child sex, preterm birth (< 37 weeks of gestation), parental education level (tertiary education vs. upper secondary or lower), maternal smoking during pregnancy (yes/no), diagnosed gestational diabetes (yes/no), and parental country of birth (both parents born outside Sweden vs. at least one parent born in Sweden).

### 1.3 Participants

The study population was drawn from the Alma cohort, including all children born in Sweden between 1 January 2018 and 31 December 2023. Children were eligible for inclusion if they had undergone the routine language screening within the Swedish child health services programme by 25 March 2025. In Sweden, language screening is routinely conducted between 2 years 4 months and 3 years 3 months of age.

Children with missing information on birth size for gestational age were excluded. Children from multiple pregnancies were excluded because the Maršál reference values were derived from singleton pregnancies and are not applicable to this group. For children with multiple screening records, the latest available screening was retained. The selection of the study population is illustrated in Figure 1.

**FIGURE 1:**
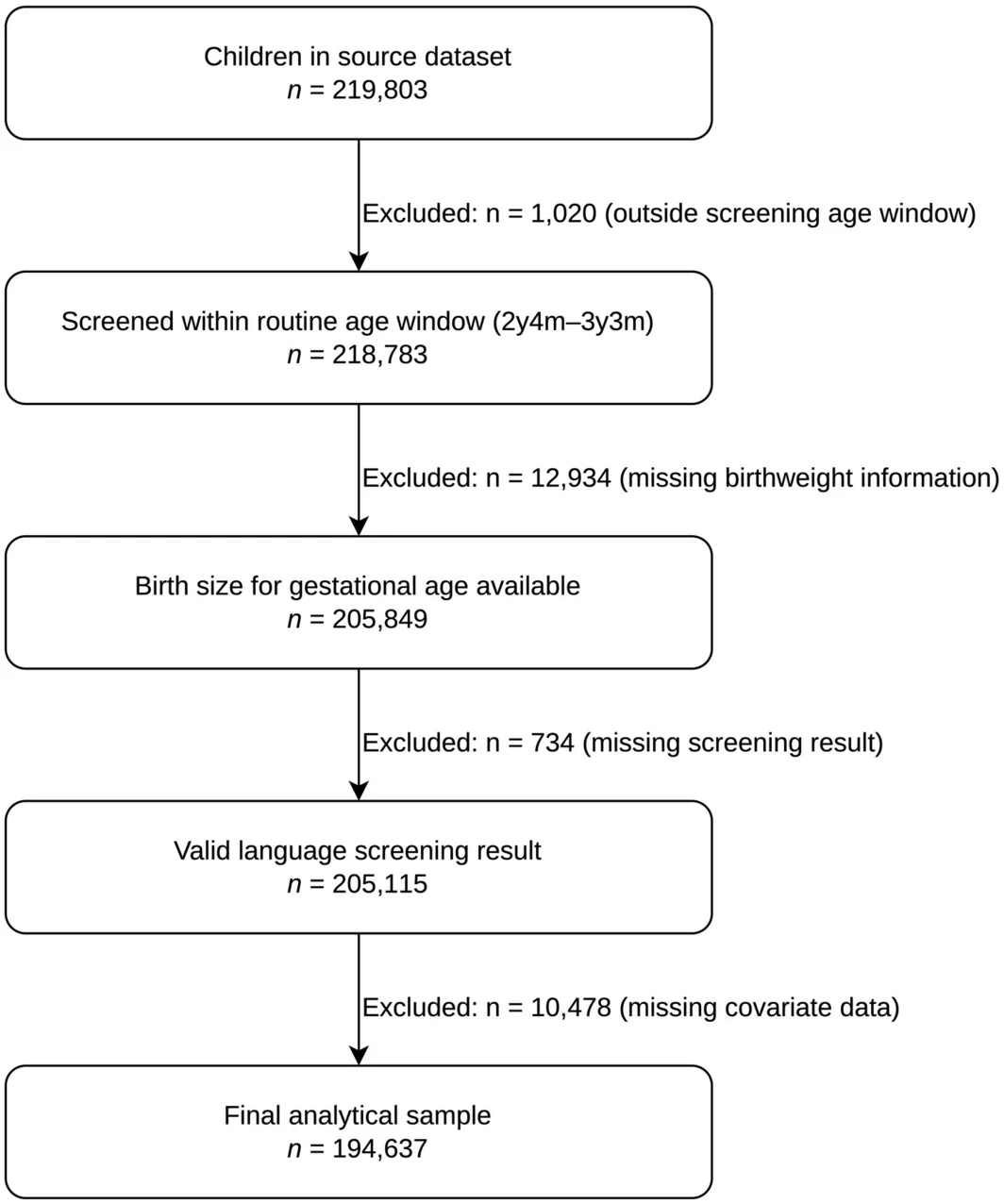
Flowchart of data exclusion.

### 1.4 Statistical Analysis

Associations between birth size for gestational age and language screening outcome were analysed using logistic regression. Separate models were estimated for children born SGA and LGA, each compared with children born AGA as the reference group.

First, crude models were fitted, including only the exposure variable. Subsequently, adjusted models were estimated controlling for potential confounding factors identified a priori: child sex, preterm birth (< 37 weeks of gestation), gestational diabetes, parental education level, maternal smoking during pregnancy, and parental country of birth. All potential confounders were entered simultaneously into a single adjusted model, consistent with standard epidemiological practice.

Analyses were conducted using complete‐case data, excluding observations with missing information on the included covariates. Results are presented as odds ratios (OR) with 95% confidence intervals (CI). Statistical analyses were performed using R. Multicollinearity among covariates was assessed using variance inflation factors, which indicated no evidence of problematic collinearity.

## 2 Results

A total of 194 637 children were included in the analyses. Of these, 4194 (2.2%) were classified as SGA and 6883 (3.5%) as LGA. Overall, 12% of the children failed the language screening.

Mean age at screening was 2 years and 9 months (SD 3.0 months), within the routine screening interval of 2 years 4 months to 3 years 3 months. Descriptive characteristics of the study population according to birth size for gestational age are presented in Table 1.

**TABLE 1:** Study population characteristics.

Characteristic	SGA *N* = 4194^a^	AGA *N* = 183560^a^	LGA *N* = 6883^a^
Language screening result			
Pass	3413 (81.4%)	162 010 (88.3%)	5935 (86.2%)
Fail	781 (18.6%)	21 550 (11.7%)	948 (13.8%)
Sex			
Girl	1997 (47.6%)	89 633 (48.8%)	3421 (49.7%)
Boy	2197 (52.4%)	93 927 (51.2%)	3462 (50.3%)
Premature	780 (18.6%)	6481 (3.5%)	400 (5.8%)
Education level			
Tertiary or higher	2539 (60.5%)	119 033 (64.8%)	4088 (59.4%)
Upper secondary	1655 (39.5%)	64 527 (35.2%)	2795 (40.6%)
Smoking			
Non‐smoker	3900 (93.0%)	177 301 (96.6%)	6703 (97.4%)
Smoker	294 (7.0%)	6259 (3.4%)	180 (2.6%)
Foreign‐born parents			
No or one parent born outside Sweden	2785 (66.4%)	142 780 (77.8%)	5800 (84.3%)
Both parents born outside Sweden	1409 (33.6%)	40 780 (22.2%)	1083 (15.7%)
Gestational diabetes	129 (3.1%)	7361 (4.0%)	847 (12.3%)

^a^ *n* (%).

Children born SGA had higher odds of failing the language screening compared with children born AGA. In the crude model, SGA was associated with increased odds of screening failure (OR 1.72, 95% CI 1.59–1.86, *p* < 0.001). After adjustment for sex, preterm birth, parental education, maternal smoking during pregnancy, and parental country of birth, the association remained significant, although somewhat attenuated (adjusted OR 1.42, 95% CI 1.30–1.54, *p* < 0.001) (Table 2).

**TABLE 2:** Association between small for gestational age (SGA) and language screening failure.

Characteristic	Crude OR			Adjusted OR		
	OR	95% CI	*p*	OR	95% CI	*p*
SGA status^a^	1.72	1.59, 1.86	< 0.001	1.42	1.30, 1.54	< 0.001
Sex^b^				2.23	2.16, 2.30	< 0.001
Premature^c^				1.45	1.36, 1.55	< 0.001
Education level^d^				1.72	1.67, 1.77	< 0.001
Smoking^e^				1.48	1.38, 1.58	< 0.001
Foreign‐born parents^f^				2.70	2.62, 2.78	< 0.001
Gestational diabetes^g^				1.40	1.32, 1.49	< 0.001

Abbreviations: CI, confidence interval; OR, odds ratio.

^a^ SGA = 1, AGA = 0.

^b^ Male = 1, Female = 0.

^c^ Preterm birth (< 37 weeks) = 1.

^d^ Tertiary or higher education = 1.

^e^ Maternal smoking during pregnancy = 1.

^f^ Two foreign‐born parents = 1.

^g^ Gestational diabetes = 1.

Children born LGA also had higher odds of failing the language screening compared with children born AGA. In the crude model, LGA was associated with a modest increase in the odds of screening failure (OR 1.20, 95% CI 1.12–1.29, *p* < 0.001). The association remained statistically significant after adjustment for the same covariates (adjusted OR 1.24, 95% CI 1.15–1.33, *p* < 0.001) (Table 3). Among the covariates, male sex, preterm birth, lower parental education level, maternal smoking during pregnancy, and having two foreign‐born parents were all associated with increased odds of failing the language screening (Tables 2 and 3).

**TABLE 3:** Association between large for gestational age (LGA) and language screening failure.

Characteristic	Crude OR			Adjusted OR		
	OR	95% CI	*p*	OR	95% CI	*p*
LGA status^a^	1.20	1.12, 1.29	< 0.001	1.24	1.15, 1.33	< 0.001
Sex^b^				2.24	2.17, 2.31	< 0.001
Premature^c^				1.46	1.37, 1.57	< 0.001
Education level^d^				1.71	1.66, 1.76	< 0.001
Smoking^e^				1.51	1.41, 1.62	< 0.001
Foreign‐born parents^f^				2.70	2.62, 2.79	< 0.001
Gestational diabetes^g^				1.39	1.31, 1.48	< 0.001

Abbreviations: CI, confidence interval; OR, odds ratio.

^a^ LGA = 1, AGA = 0.

^b^ Male = 1, Female = 0.

^c^ Preterm birth (< 37 weeks) = 1.

^d^ Tertiary or higher education = 1.

^e^ Maternal smoking during pregnancy = 1.

^f^ Two foreign‐born parents = 1.

^g^ Gestational diabetes = 1.

## 3 Discussion

In this large population‐based study, both SGA and LGA were associated with an increased likelihood of failing routine language screening at 2.5–3 years of age. The association was stronger for children born SGA but remained statistically significant for both groups after adjustment for sex, preterm birth, parental education, maternal smoking during pregnancy, gestational diabetes and parental country of birth. These findings suggest that deviations from typical foetal growth, both restricted and excessive, may be linked to early language development. Our results extend previous research that has primarily focused on SGA by also demonstrating an association between LGA and early language screening outcomes in a large national cohort.

The proportion of infants born SGA was low (2%) in relation to international research [23], which may be explained by the higher prevalence of overweight and obesity (30%) compared to underweight (4%) among pregnant Swedish women [24], as both conditions are known to influence foetal growth. Additional explanations for the low prevalence of SGA may include Sweden's well‐functioning maternal health care system, in which almost all pregnant women receive regular antenatal monitoring. Finally, the observed prevalence of SGA may be attributable to the use of Maršál's reference values, defining SGA as below −2 SD.

The attenuation of the SGA association after adjustment, from a crude OR of 1.72 to an adjusted OR of 1.42, is consistent with partial confounding by the included covariates. Children born SGA in this cohort had higher proportions of maternal smoking and two foreign‐born parents compared with AGA children, both of which are independently associated with screening failure. That a substantial association remained after adjustment suggests that birth size itself, rather than these co‐occurring risk factors alone, contributes to the observed differences in screening outcomes.

For LGA, the association was largely stable across crude and adjusted models (OR 1.20 and 1.24, respectively), indicating that the included covariates did not substantially change the association. The modest magnitude of both the crude and adjusted estimates, combined with the large sample size, means that these findings are statistically precise but should be interpreted in terms of their clinical relevance rather than their statistical significance alone.

While prior research has documented associations between gestational diabetes and DLD [5], the present findings suggest a broader picture. Irrespective of gestational diabetes status, both LGA and SGA births appear to be factors worthy of consideration in relation to children's language development.

Children with both parents born outside Sweden had 2.7 times higher odds of failing the language screening, which may indicate that screening was not conducted according to the manual, which stipulates that children should be assessed in their dominant language. This is consistent with previous research demonstrating that bilingual children in Sweden are frequently screened in Swedish only [25].

This knowledge may enable child health services to monitor children's language development more closely and provide health promoting interventions within their mandate, potentially reducing risks for the child.

The large sample size confers considerable statistical power, such that even modest effect sizes are estimated with precision. With nearly 195 000 participants, many associations, including potentially spurious ones, are likely to reach statistical significance. This is particularly evident in the LGA model, where all covariates were highly statistically significant despite the combined effect being modest in magnitude. In this context, it is worth noting that odds ratios can be interpreted directly as measures of effect size: an adjusted OR of 1.24 for LGA indicates a 24% increase in the odds of screening failure, which, while statistically precise, represents a small effect at the individual level. Therefore, the statistical significance of findings in this study should not be interpreted as evidence of strong or clinically meaningful associations, and readers are encouraged to focus on the magnitude and confidence intervals of the odds ratios rather than on *p*‐values. At a population level, given the high prevalence of language screening failure overall (12%), even a relatively small increase in odds translates to a meaningful number of affected children. At an individual level, however, SGA or LGA status alone is a weak predictor of screening failure and should be considered alongside other known risk factors rather than in isolation.

A limitation of the study is that SGA and LGA were classified using the reference values by Maršál et al. [7]. Although the −2 SD and +2 SD thresholds are standard in Swedish clinical practice, a recent study on a Swedish population found that the WHO standards [26] provided a better fit for classifying full‐term infants, reducing low sensitivity and high false‐positive rates [27]. Alternative classification approaches include percentile‐based thresholds (commonly the 10th and 90th percentiles) and international standards such as the Intergrowth‐21st foetal growth standards [28].

A further limitation is that two different validated screening instruments are used across Swedish regions, which may introduce some heterogeneity in how pass/fail outcomes are assigned, despite both instruments assessing the same core language functions.

The analysis was limited to screening outcomes and did not include formal language assessments or a diagnosis of DLD. Future studies should investigate the relationship between language screening outcomes and later DLD diagnoses, as well as the associations between SGA, LGA, and DLD. An additional limitation of the present study is the absence of data regarding parental history of DLD. As there is a well‐established genetic component to DLD [29], the lack of such information may have influenced the results.

Multiple births were excluded for several reasons, including the fact that the Maršál reference values do not apply to this group and that multiple gestations involve distinct inutero risk profiles. Consequently, the findings are not generalizable to children born from multiple births.

## 4 Conclusion

In this large population‐based cohort study, both SGA and LGA were independently associated with increased odds of failing routine language screening at approximately 2.5–3 years of age. The association was stronger for SGA than for LGA, and both remained statistically significant after adjustment for established risk factors including sex, preterm birth, parental education, maternal smoking, gestational diabetes, and parental country of birth. These findings suggest that deviations from typical foetal growth in either direction may carry implications for early language development, extending prior research that has largely focused on SGA alone.

Given the high overall prevalence of screening failure in this cohort and the independent contribution of birth size for gestational age, these results may have relevance for clinical practice within child health services. Awareness of SGA and LGA status could inform more targeted monitoring of language development in early childhood, though SGA or LGA alone should be interpreted as one risk indicator among several rather than as a strong individual predictor.

Future research should examine the relationship between birth size for gestational age and formally diagnosed developmental language disorder, and investigate the extent to which early identification and intervention may modify outcomes for children in these groups.

## Author Contributions

**Anna Fäldt:** conceptualization, investigation, funding acquisition, methodology, formal analysis, writing – review and editing, writing – original draft, resources. **Isabelle Liljekvist:** writing – original draft, writing – review and editing, investigation, formal analysis. **Anton Dahlberg:** conceptualization, investigation, funding acquisition, writing – original draft, methodology, validation, writing – review and editing, software, supervision, data curation, formal analysis, resources, project administration.

## Funding

This work was supported by Forskningsrådet om Hälsa, Arbetsliv och Välfärd, 2023‐01130.

## Conflicts of Interest

The authors declare no conflicts of interest.

## Data Availability

The data that support the findings of this study are available on request from the corresponding author. The data are not publicly available due to privacy or ethical restrictions.
